# Theoretical investigations on modeling blood flow through vessel for understanding effectiveness of magnetic nanocarrier drug delivery systems

**DOI:** 10.3389/fmed.2024.1397648

**Published:** 2024-05-22

**Authors:** Rami M. Alzhrani, Mohammed F. Aldawsari, Jawaher Abdullah Alamoudi

**Affiliations:** ^1^Department of Pharmaceutics and Industrial Pharmacy, College of Pharmacy, Taif University, Taif, Saudi Arabia; ^2^Department of Pharmaceutics, College of Pharmacy, Prince Sattam Bin Abdulaziz University, Al-Kharj, Saudi Arabia; ^3^Department of Pharmaceutical Sciences, College of Pharmacy, Princess Nourah bint Abdulrahman University, Riyadh, Saudi Arabia

**Keywords:** drug delivery, cancer therapy, nanocarrier, decision tree, multilayer perceptron

## Abstract

For cancer therapy, the focus is now on targeting the chemotherapy drugs to cancer cells without damaging other normal cells. The new materials based on bio-compatible magnetic carriers would be useful for targeted cancer therapy, however understanding their effectiveness should be done. This paper presents a comprehensive analysis of a dataset containing variables *x*(m), *y*(m), and *U*(m/s), where *U* represents velocity of blood through vessel containing ferrofluid. The effect of external magnetic field on the fluid flow is investigated using a hybrid modeling. The primary aim of this research endeavor was to construct precise and dependable predictive models for velocity, utilizing the provided input variables. Several base models, including K-nearest neighbors (KNN), decision tree (DT), and multilayer perceptron (MLP), were trained and evaluated. Additionally, an ensemble model called AdaBoost was implemented to further enhance the predictive performance. The hyper-parameter optimization technique, specifically the BAT optimization algorithm, was employed to fine-tune the models. The results obtained from the experiments demonstrated the effectiveness of the proposed approach. The combination of the AdaBoost algorithm and the decision tree model yielded a highly impressive score of 0.99783 in terms of *R*^2^, indicating a strong predictive performance. Additionally, the model exhibited a low error rate, as evidenced by the root mean square error (RMSE) of 5.2893 × 10^−3^. Similarly, the AdaBoost-KNN model exhibited a high score of 0.98524 using *R*^2^ metric, with an RMSE of 1.3291 × 10^−2^. Furthermore, the AdaBoost-MLP model obtained a satisfactory *R*^2^ score of 0.99603, accompanied by an RMSE of 7.1369 × 10^−3^.

## 1 Introduction

There are different challenges in medical science among which cancer therapy is one of the most major challenges which is currently being studied to improve the efficiency of treatment (1, 2). For cancer therapy, the drug must reach the cancer cell at desired dosage, while other adjacent normal cells should be intact. Indeed, targeted cancer therapy would be of great importance for scientists to develop drug delivery systems with high efficacy (3, 4). Recently, bio-compatible magnetic nanocarriers have attracted much attention due to their superior properties in cancer treatment (5–7). The motion of these particles in the blood circulating system can be monitored and controlled using magnetic field such as external permanent magnet (8, 9).

Computational methods can be developed and implemented for magnetic-based drug delivery systems to understand the effectiveness of drug as well as the targeting the drug-carrier formulation. For modeling, fluid mechanics as well as magnetic models should be combined to build a holistic model of system (10, 11). For model development, the interactions between the nanoparticles and the medium as well as the external force should be taken into account. Since the fluid flow contains magnetic nanoparticles, ferrohydrodynamics of blood would help one understand the flow pattern and monitor the agglomeration of nanoparticles in the blood stream. Indeed, the agglomeration of drug loaded nanoparticles would cause problems and reduce the efficacy of targeted cancer treatment.

Among different modeling approaches developed for drug delivery systems, the models based on fluids mechanics have shown great capability in which the blood flow in vessel can be modeled by solution of fluid mechanics equations (12). The flow in this case is considered to be under the low Reynolds number flow regime. Therefore, the main forces applied on the fluid flow are magnetic force as well as viscous forces (12). So, numerical solution of the governing equations is the main methodology for simulation of blood flow with magnetic nanoparticles. However, other modeling strategies can be integrated to the fluid mechanic models to facilitate the simulation, such as machine learning models which have attracted much attention in different fields. The methods of machine learning have been used with integration to computational fluid mechanics (CFD) models to reduce the complexity of CFD models (13). This approach can be also adopted in this work for modeling targeted cancer therapy using magnetic nanoparticles.

The field of machine learning (ML) has proven to be highly effective in uncovering intricate patterns within complex systems and making accurate predictions based on input variables (14, 15). These techniques have revolutionized various domains, including data analysis, artificial intelligence, and predictive modeling. By leveraging ML algorithms and models, researchers and practitioners can extract valuable insights from vast amounts of data. ML techniques excel at identifying hidden relationships and patterns that may not be immediately apparent to human observers. This ability to capture intricate patterns empowers ML models to make precise predictions and inform decision-making processes. A wide range of ML models have been developed and utilized in science and engineering.

The main objective of this research was to develop accurate and reliable predictive ML models for velocity simulation of blood flow using the given input variables. Various foundational models, such as K-nearest neighbors (KNN), decision tree (DT), and multilayer perceptron (MLP), were trained and assessed. Furthermore, an ensemble model known as AdaBoost was employed to augment the predictive performance. The models are integrated to CFD model developed for simulation of blood flow in vessel for targeted cancer therapy.

K-nearest neighbors (KNN) is a non-parametric classification and regression algorithm that assigns a data point to a class based on the majority class among its K-nearest neighbors (16). While versatile, it faces challenges like the curse of dimensionality. Decision trees (DT) construct tree-shaped models for decision-making, offering interpretability but prone to overfitting and sensitivity to minor data variations (17). Multilayer perceptron (MLP), with interconnected layers of neurons, excel in pattern recognition but encounter computational challenges and local optima during training (18). AdaBoost combines weak models, transferring gradients to improve accuracy, adapting well to noisy data and outliers while leveraging knowledge from previous estimators for enhanced performance.

Employing individual machine learning methods in their basic forms may lead to models with reduced accuracy or potential overfitting. In this research, we hypothesized that utilizing AdaBoost and BAT algorithm to regulate hyperparameters and modulate the complexity of models could yield more precise and generalized models. This hypothesis was validated through the analysis of results.

## 2 Materials and methods

### 2.1 Data set description and model

**Figure 1:**
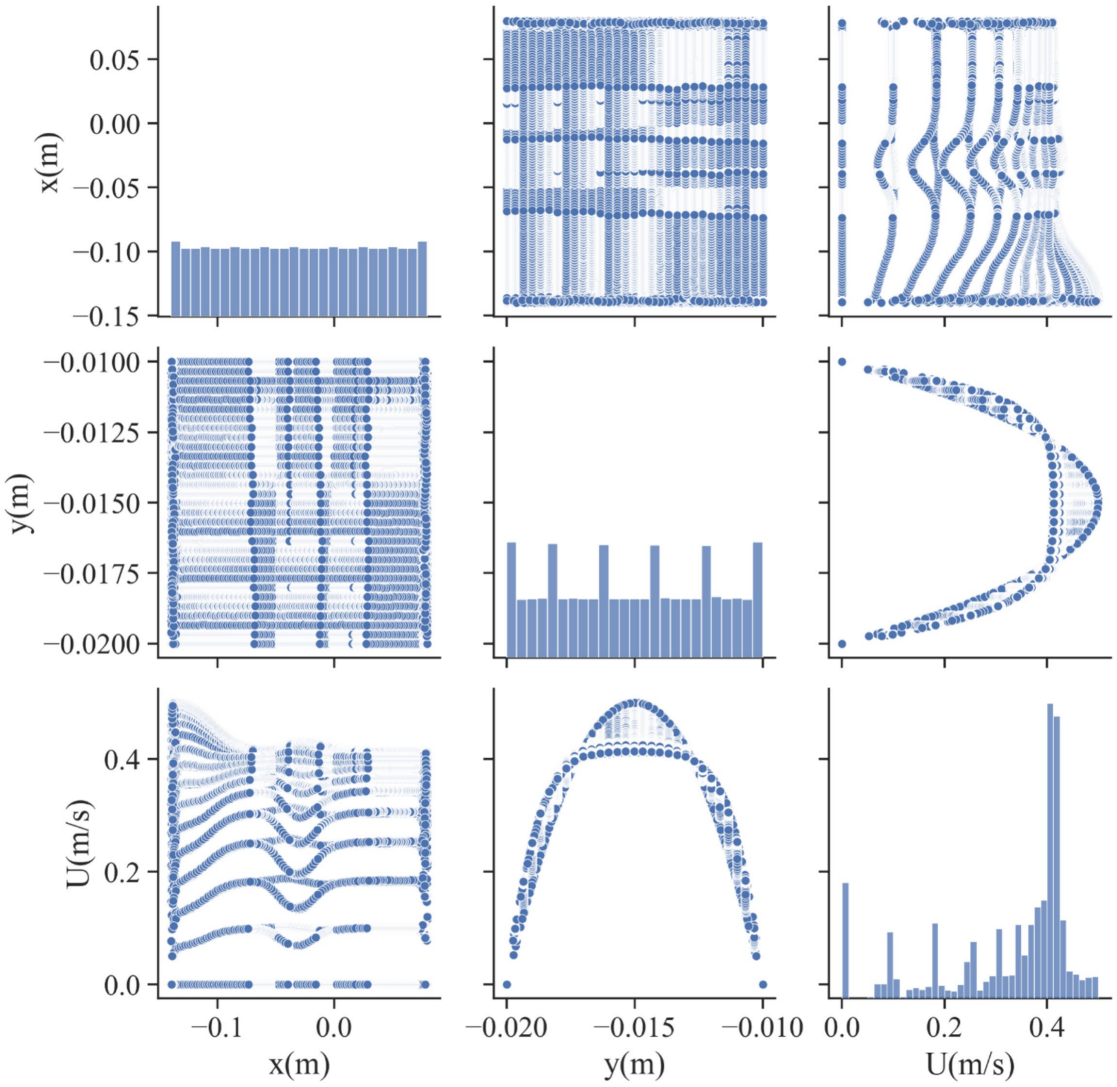
Pair plot of the dataset for velocity field, *U*.

**Figure 2:**
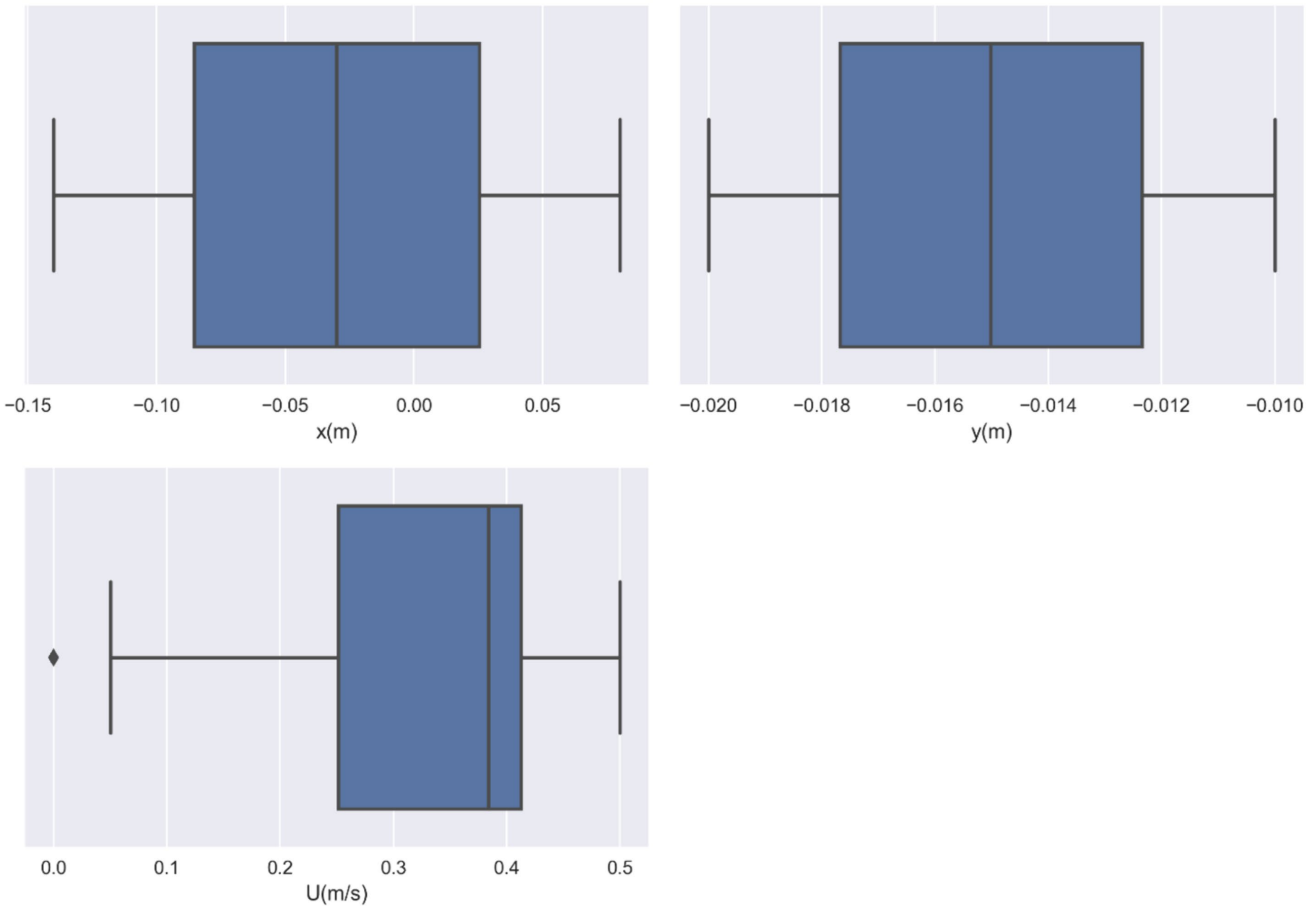
Variable box plots for the input and output parameters.

In this study, the dataset used consists of more than 17,000 data entries. The dataset’s structure involves utilizing spatial coordinates as input, while the output variable is represented as the velocity, indicated as *U*, and measured in meters per second (m/s). *U*, as the only response, is indeed the velocity field for blood flow through vessel which contain drug loaded magnetic nanoparticles. The influence of external magnetic force on the nanoparticles was considered in the CFD model development. The obtained velocity field in 2 dimensional (2D) using the CFD was then used for training/testing the ML models. Therefore, the model is built in two steps, i.e., CFD simulation and ML development using the CFD outputs. The description of the system containing vessel and the external magnet has been reported elsewhere (19). The pairwise distribution of the dataset variables is visualized in Figure 1 and the box plots are depicted in Figure 2. Also Table 1 shows the statistics of the dataset.

**Table 1:** Statistical measures of dataset variables.

Statistic	*X* (m)	*Y* (m)	*U* (m/s)
Mean	−0.029661	−0.015008	0.338124
Standard deviation	0.062820	0.002845	0.114155
Minimum	−0.140000	−0.020000	0.000000
Maximum	0.080000	−0.010000	0.500050

The main equation which is solved via numerical technique is the Navier–Stokes equations which are solved via finite element method (FEM). The equations can be expressed as:


$$\rho \frac{\partial U}{\partial t}-\nabla \cdot \mu \left(\nabla U+{\left(\nabla U\right)}^{T}\right)+\rho U\cdot \nabla U+\nabla p=F$$

∇·U=0

where *U* is velocity of blood through the vessel (m/s). *p* is the pressure, and *F* is body force (*N*). *F* as the body forces is estimated using the external magnetic field based on Maxwell’ model (19). The geometry of the vessel is considered as a tubular shape with axial symmetry where the input velocity is considered to be the well-known parabolic velocity profile. Additionally, a sinusoidal velocity profile was considered to take into account the influence of hear beat on the velocity profile. The simulations in this study were performed for the case at a hear beat.

In this study, prior to delving into the execution of regression analysis, sophisticated preprocessing steps were undertaken to ensure the integrity and reliability of the dataset. Beginning with the identification and isolation of outliers through the innovative Isolation Forest algorithm, the data underwent a rigorous cleansing process, effectively eliminating anomalous data points that could potentially skew the subsequent regression analysis. Following outlier detection, the dataset was subjected to min-max normalization, a transformation technique aimed at standardizing the range of values across different features, thereby enhancing the stability and interpretability of the regression model. Furthermore, to gauge the model’s performance accurately, the dataset was judiciously partitioned into training and testing subsets, with an 80-20 ratio, ensuring a robust evaluation framework. Through this comprehensive preprocessing pipeline, the study lays a solid foundation for rigorous regression analysis, poised to uncover meaningful insights with confidence and precision.

### 2.2 Base regression models

After the simulation of fluid flow using the Navier–Stokes equations, the velocity distribution in two dimensional were obtained and extracted for ML model development. A number of ML models were used which will be explained in the following sections. Therefore, the ML models possess two inputs which are the coordinates and sole response, which is the velocity field, *U* (m/s).

The decision tree (DT) model is a highly advantageous option for regression applications. The technique is dependent on a data structure resembling a tree and functions as a means for selecting features optimally during the process of dividing the tree. The core aim is to identify a feature that effectively refines the split dataset and brings order to the initially disordered data. The decision tree model comprises three fundamental components: root nodes, intermediate nodes, and leaf or terminal nodes. The leaf nodes indicate the ultimate predictions, while the remaining nodes serve as evaluative points for various attributes. At each node, data samples are subdivided into child nodes (sub-nodes) based on property tests and the resultant insights (20, 21).

All data points are inputted into the root node during the initial stage of the training phase. Subsequently, the DT algorithm determines the optimal strategy for dividing the data into partitions. As this method is applied, each sub-dataset resulting from the division adheres to the criteria of the division rule, ensuring that all samples are appropriately categorized. The initial dataset undergoes successive subdivisions until a tree-like structure is formed, giving rise to several meticulously refined datasets (22).

The attainment of optimal “purity” in a DT occurs when all instances within a specific branch node are classified into the same category, as previously mentioned. Various metrics are commonly employed to assess the purity of divided samples and examine the integrity of datasets. The metrics encompassed in this set are information gain ratio, entropy, and the Gini index (23).

Information entropy measures dataset impurity, reflecting uncertainty in sample distribution across categories. Decision tree algorithms leverage entropy to assess information gain from attribute-based data splits. Gain ratio evaluates split effectiveness, considering both information gain and intrinsic attributes’ information to mitigate biases from attributes with many distinct values. The Gini index, akin to entropy, gauges impurity by the probability of misclassifying a randomly chosen sample. Decision trees use the Gini index to identify optimal attribute splits minimizing impurity in branch nodes. Employing these metrics, decision tree algorithms ensure accurate and informative model construction, selecting attribute splits that enhance tree purity and effectiveness.

Another foundational model utilized in this research is the K-nearest neighbors (KNN) model. The KNN technique is rooted in non-parametric and similarity-based learning methods (24). These methods do not impose any assumptions about the data distribution. The KNN algorithm belongs to the realm of supervised machine learning techniques and stands out for its simplicity, straightforward implementation, and the absence of a substantial training time requirement. This versatility makes it applicable to addressing both classification and regression challenges (25, 26).

In this approach, the identification of nearest neighbor locations relies on distance metrics, commonly utilizing Euclidean distance. However, other distance metrics like Manhattan distance, Minkowski distance, and various alternatives are also employed. The Euclidean distance is calculated using the following equation (27):


$$D\left(X,Y\right)=\sqrt{\overset{k}{\underset{i=1}{\sum }}{\left(X_{i}-Y_{i}\right)}^{2}}$$

Multilayer perceptron regression (MLP) stands as an impressive and versatile machine learning model, capable of tackling both regression and classification tasks with finesse. This model belongs to the esteemed family of feedforward artificial neural networks, drawing inspiration from the intricate workings of the human brain.

Within the realm of regression, MLP boasts a sophisticated multilayer architecture comprising an input layer, one or multiple hidden layers, and an output layer. These layers house artificial neurons, or nodes, interconnected through weighted connections, forging a pathway for information flow.

At the heart of MLP lies the mission to comprehend intricate, nonlinear relationships within the data. This journey commences with the transformation of input features, traversing a realm of weighted computations and activation functions, ultimately yielding a predicted output. This intricate process is mathematically encapsulated as follows (28):


$$\begin{array}{l} y=f(W^{\left(L\right)}f(W^{\left(L-1\right)}(...f(W^{\left(2\right)}f(W^{(1)x}\\ \;\;\;\;\;\;\;\;\;+b^{\left(1\right)})+b^{\left(2\right)})...)+b^{\left(L-1\right)}+b^{\left(L\right)}) \end{array}$$

Here, x stands for the illustrious input features, $W^{\left(i\right)}$ symbolizes the weight matrix of layer *i,* and $b^{\left(i\right)}$ embodies the captivating bias vector of layer *i*. Also, *f* signifies the activation function, often a mesmerizing nonlinear entity such as ReLU (Rectified Linear Unit) or sigmoid.

MLP embarks on a journey of enlightenment by minimizing an aptly chosen loss function (e.g., MSE) through an optimization algorithm like the enchanting gradient descent. This iterative voyage adjusts the weights and biases, unlocking the true potential of the model’s predictive prowess. The learning rate ( η ) gracefully guides the magnitude of the weight updates.

The remarkable flexibility and capacity of MLP to capture intricate, nonlinear relationships make it an invaluable instrument for an array of regression tasks. From the captivating realm of financial forecasting to the captivating art of image analysis and the captivating mastery of natural language processing, MLP shines as a beacon of hope. With the right blend of adaptability and meticulous hyperparameter tuning, MLP emerges as a reliable and accurate solution, transcending barriers to conquer real-world regression challenges.

### 2.3 AdaBoost method

Adaptive boosting, also known as AdaBoost, is a technique that utilizes a group of multiple base models, with each model performing slightly better than a random predictor. The key concept behind AdaBoost is the transfer of gradients from preceding base estimators to subsequent ones, enabling the minimization of inaccuracies in previous models and enhancing their overall accuracy (29).

Through the sequential learning process, each base estimator in AdaBoost builds upon the knowledge of its predecessors, resulting in an increase in the overall cognitive ability of the learner. This sequential learning approach allows AdaBoost to effectively handle outliers and noisy data, making it a robust technique for predictive modeling.

The final prediction in AdaBoost is determined by combining the estimates from all the individual weak models using weighted averaging. This ensemble approach leverages the collective knowledge of the weak models to generate a more accurate and robust prediction.

**Figure 3:**
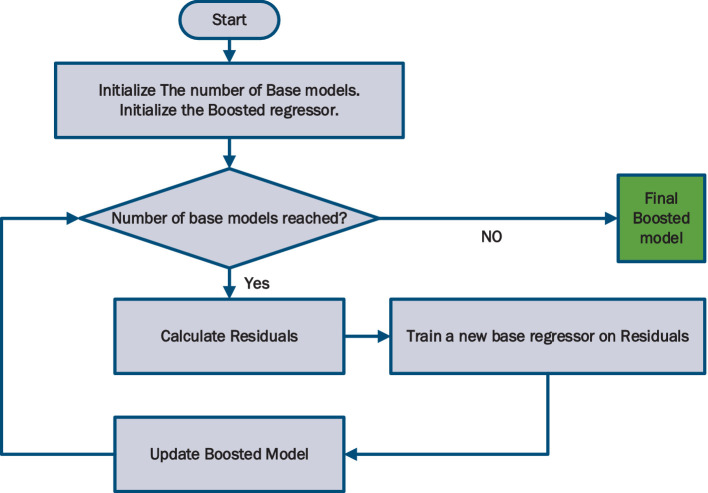
Flowchart of AdaBoost method.

The flowchart of Figure 3 shows the workflow of AdaBoost. One of the advantages of AdaBoost is its adaptability, as it can effectively handle difficult-to-predict training examples by focusing the attention of subsequent base estimators on such instances. This adaptive nature allows AdaBoost to continually improve its performance and make accurate predictions.

### 2.4 BAT optimization algorithm for hyperparameter tuning

In recent years, the optimization of hyperparameters has become a critical aspect of enhancing the performance of machine learning models. The BAT (Bat Algorithm) optimization algorithm, inspired by the echolocation behavior of bats, presents an innovative approach to efficiently tuning hyperparameters.

The Bat Algorithm, simulates the echolocation behavior of bats to find optimal solutions in a search space. It is particularly well-suited for optimization problems due to its ability to balance exploration and exploitation effectively (30).

The algorithm introduces a population of virtual bats, each representing a potential solution in the hyperparameter space. These bats fly through the search space while adjusting their frequencies and loudness, mimicking the echolocation characteristics observed in real bats. The algorithm optimizes the solutions based on the fitness of each bat in the given problem context.

The application of BAT algorithm to hyperparameter tuning involves formulating the optimization problem specific to the model and dataset at hand. Let Θ represent the hyperparameter vector, and f(Θ) denote the objective function to be optimized.

The BAT algorithm updates the position of each bat in the hyperparameter space using the following equations (31):

- Frequency:

$$f_{i}=f_{\min}+\left(f_{\max}-f_{\min}\right)\times \beta$$

- Velocity:

$$v_{i}=v_{i}+\left(\Theta_{i}-\Theta_{\mathrm{bat}}\right)\times f_{i}$$

- New position:

$$\Theta_{i}=\Theta_{i}+v_{i}$$

Here, $f_{i}$ represents the frequency of the bat, $v_{i}$ is the velocity, β is a random vector, and $\Theta_{\mathrm{bat}}$ denotes the best solution found so far.

The BAT optimization algorithm for hyperparameter tuning offers several advantages. It explores the hyperparameter space efficiently, adapts to different problem landscapes, and is capable of escaping local optima. However, practitioners should consider the sensitivity of the algorithm to the choice of parameters, such as the population size and the range of frequencies.

In a nutshell the utilization of the BAT optimization algorithm for the purpose of hyperparameter tuning presents a novel and bio-inspired methodology aimed at improving the efficacy of machine learning models. The BAT algorithm’s ability to effectively explore the hyperparameter space makes a significant contribution to the ongoing advancement of optimization techniques within the field.

## 3 Results and discussion

**SCHEME 1:**
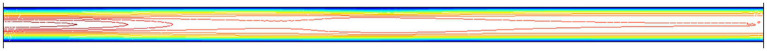
Contour of velocity field in 2 dimensions, obtained by CFD simulations.

In this study, we investigated the relationship between variables *x*(m), *y*(m), and *U*(m/s), where *U* represents the velocity. We employed several base models, namely decision tree (DT), K-nearest neighbors (KNN), and multilayer perceptron (MLP), to predict the velocity based on the given variables. Additionally, we utilized the ensemble model AdaBoost to enhance the predictive performance of our models. In the first step of modeling, CFD simulations were conducted to obtain the velocity field for the blood flow through the vessel. The results of CFD for velocity field are illustrated in Scheme 1. As seen, the velocity tends to zero near the wall as no-slip boundary condition was used for the wall. Moreover, the parabolic velocity profile can be seen in the vessel which resembles the flow through a tube. The maximum velocity occurs at the center of the blood vessel.

**Figure 4:**
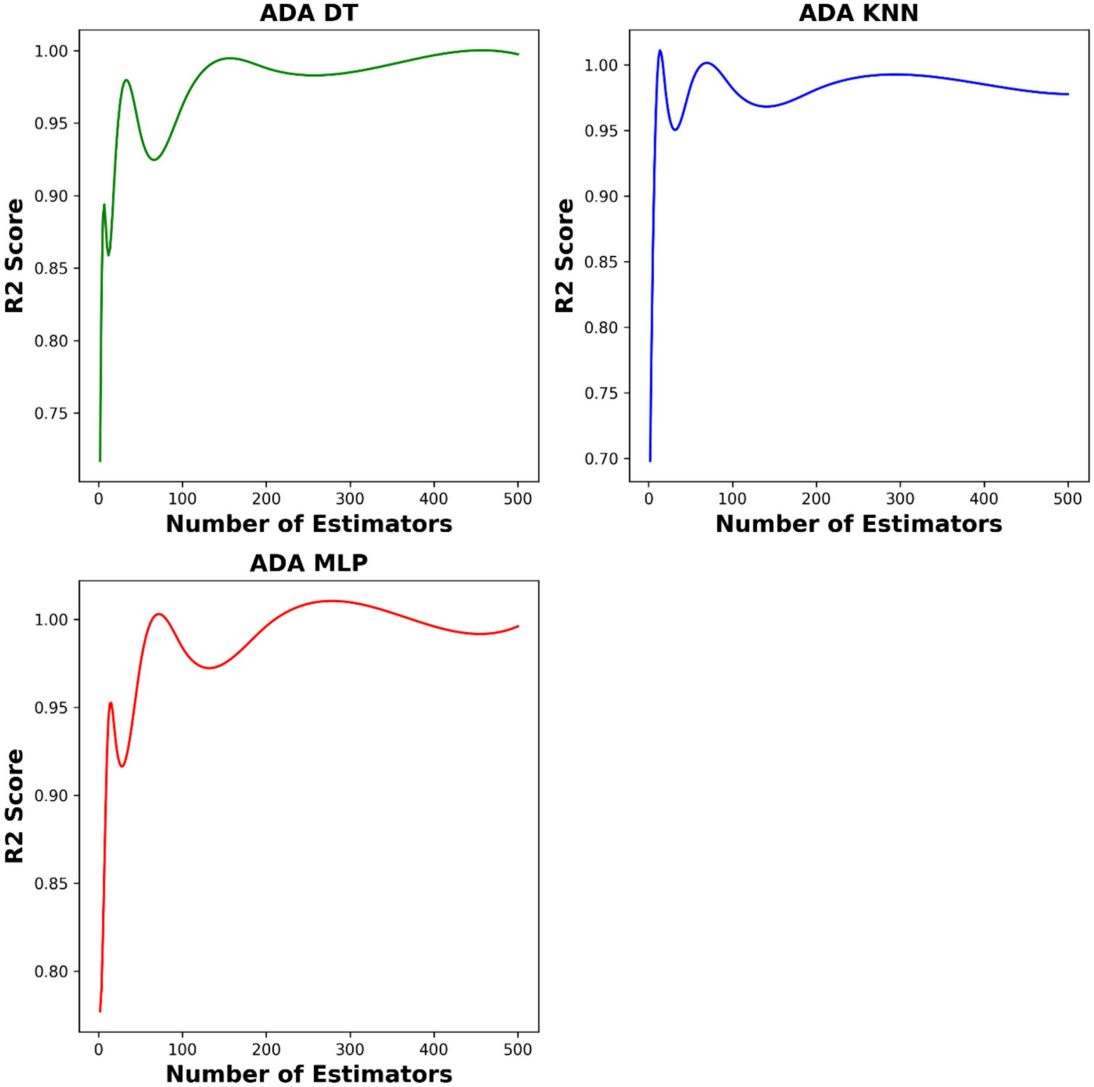
The impact of number of base models on the model performances.

To fine tune the hyper-parameters of our models, we employed the BAT optimization algorithm. This algorithm efficiently explores the parameter space and finds the optimal values, leading to improved model performance. The critical hyper-parameters include setting the optimal value of K to 2 for the base K-nearest neighbors (KNN) model, determining a maximum tree depth of 8 for decision trees (DT), and selecting hidden layer sizes of (66, 29) for the multi-layer perceptron (MLP). Additionally, the number of estimators in AdaBoost on top of the base models are 10, 180, and 450 for ADA-KNN, ADA-MLP, and ADA-DT, respectively. Figure 4 illustrates the influence of the number of estimators on model performances.

The results obtained from our experiments are presented in Table 2. We evaluated the performance of each model in terms of the *R*^2^ score and the RMSE.

**Table 2:** Performance of base models and AdaBoost ensemble.

Model	*R*^2^ score	RMSE
ADA-DT	0.99783	5.2893 × 10^−3^
ADA-KNN	0.98524	1.3291 × 10^−2^
ADA-MLP	0.99603	7.1369 × 10^−3^

**Figure 5:**
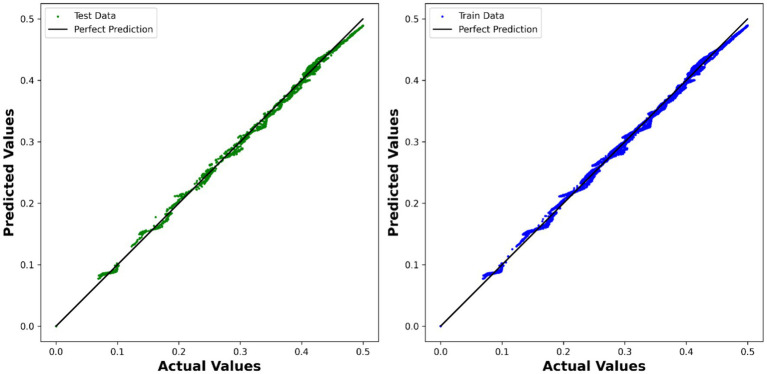
Visualization of expected values compared to predicted values using ADA-DT model.

Our results indicate that all base models, when combined with AdaBoost, achieved high accuracy in predicting the velocity. The ADA-DT model demonstrated outstanding performance, exhibiting a significant *R*^2^ score of 0.99783 and a low RMSE of 5.2893 × 10^−3^. This suggests that the decision tree-based model, when boosted with AdaBoost, effectively captured the underlying patterns in the data and produced accurate predictions. The comparison between the expected values and the predicted values is depicted in Figure 5 using the model.

**Figure 6:**
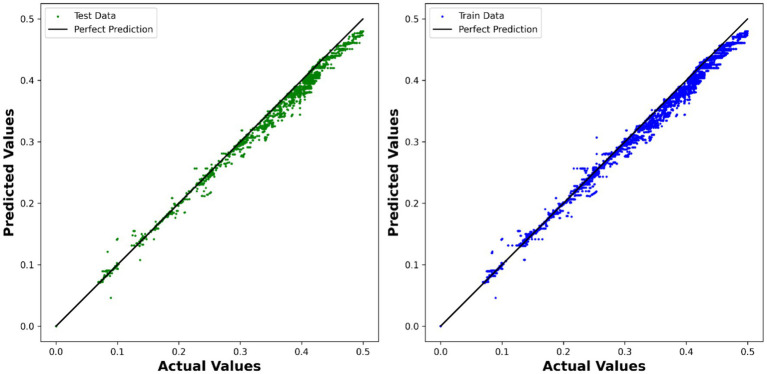
Visualization of expected values compared to predicted values using ADA-KNN model.

The ADA-KNN model also yielded promising results, with an *R*^2^ test score of 0.98524 and an RMSE of 1.3291 × 10^−2^. This indicates that the K-nearest neighbors algorithm, in conjunction with AdaBoost, successfully captured the local relationships in the dataset and provided accurate predictions. This model is used to depict the comparison between the expected and predicted values in Figure 6.

**Figure 7:**
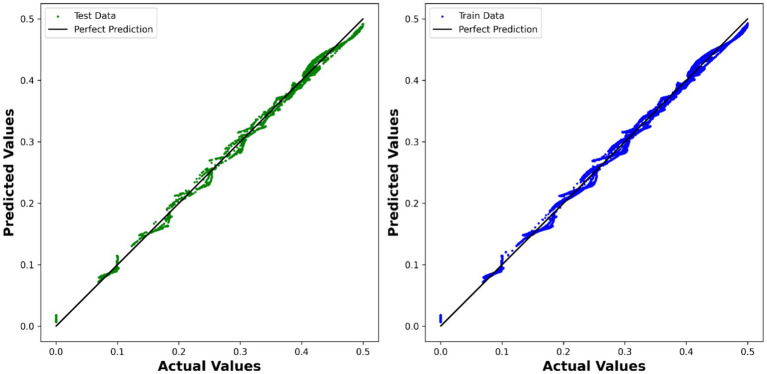
Visualization of expected values compared to predicted values using ADA-MLP model.

Similarly, the ADA-MLP model demonstrated strong predictive performance, with an *R*^2^ test score of 0.99603 and an RMSE of 7.1369 × 10^−3^. This suggests that the multilayer perceptron, coupled with AdaBoost, effectively learned the complex relationships between the variables and accurately predicted the velocity. The comparison between the expected values and the predicted values is depicted in Figure 7 using the model.

**Figure 8:**
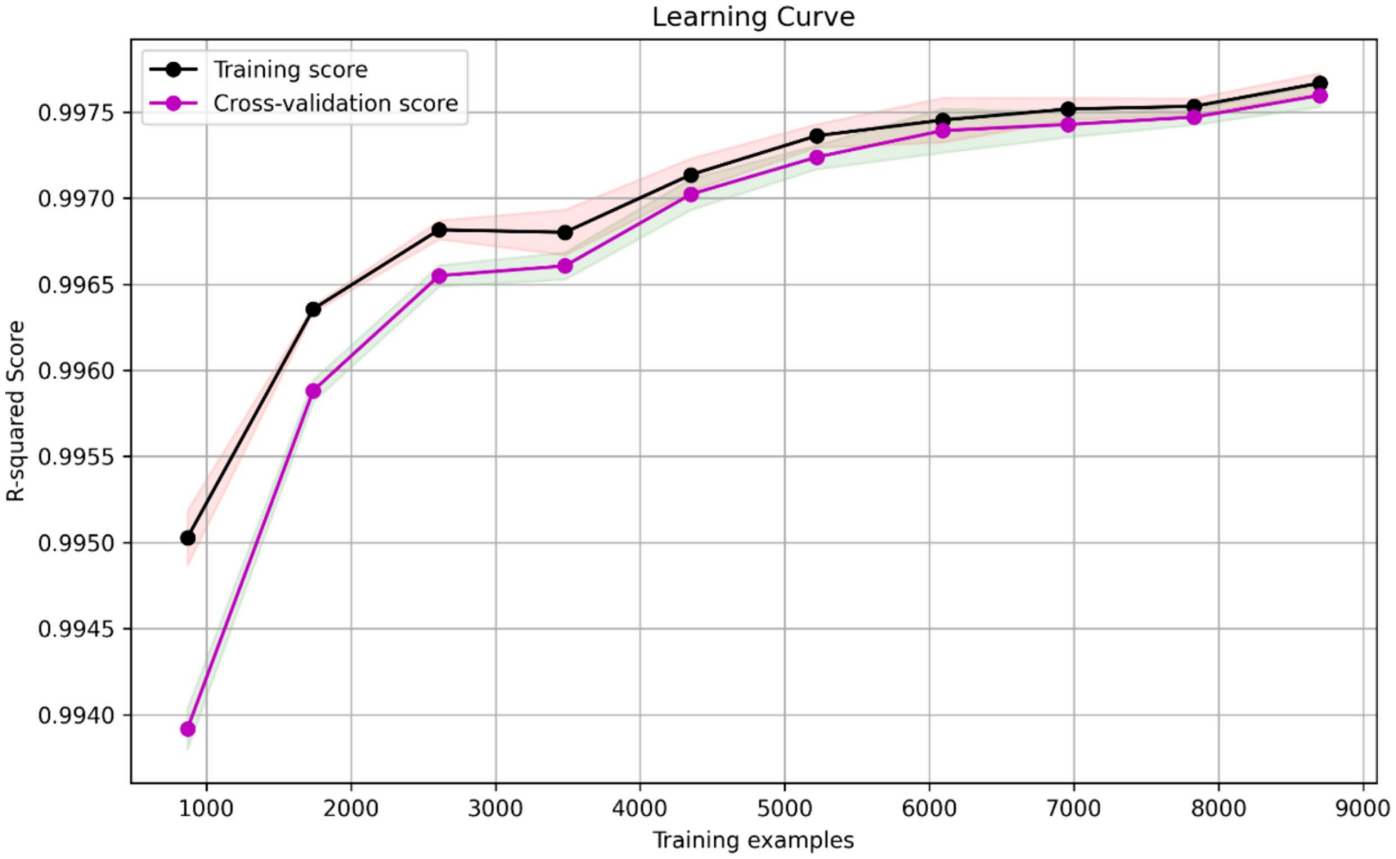
Learning curve for ADA-DT model.

Overall, our findings highlight the effectiveness of AdaBoost in improving the performance of base models in predicting the velocity based on variables *x*(m) and *y*(m). The high *R*^2^ scores and low RMSE values obtained demonstrate the accuracy and reliability of our models. The ADA-DT performs relatively better than the two other models. The learning curve depicted in Figure 8, serves as a validation of the ADA-DT performance. It illustrates how the model’s training, and cross-validation scores evolve with an increasing number of training examples. Notably, as the number of training examples grows, both scores converge and stabilize, indicating that the model generalizes well to unseen data. This convergence suggests that the model is effectively learning from the training data and can make reliable predictions on new instances, affirming its validity and robustness.

**Figure 9:**
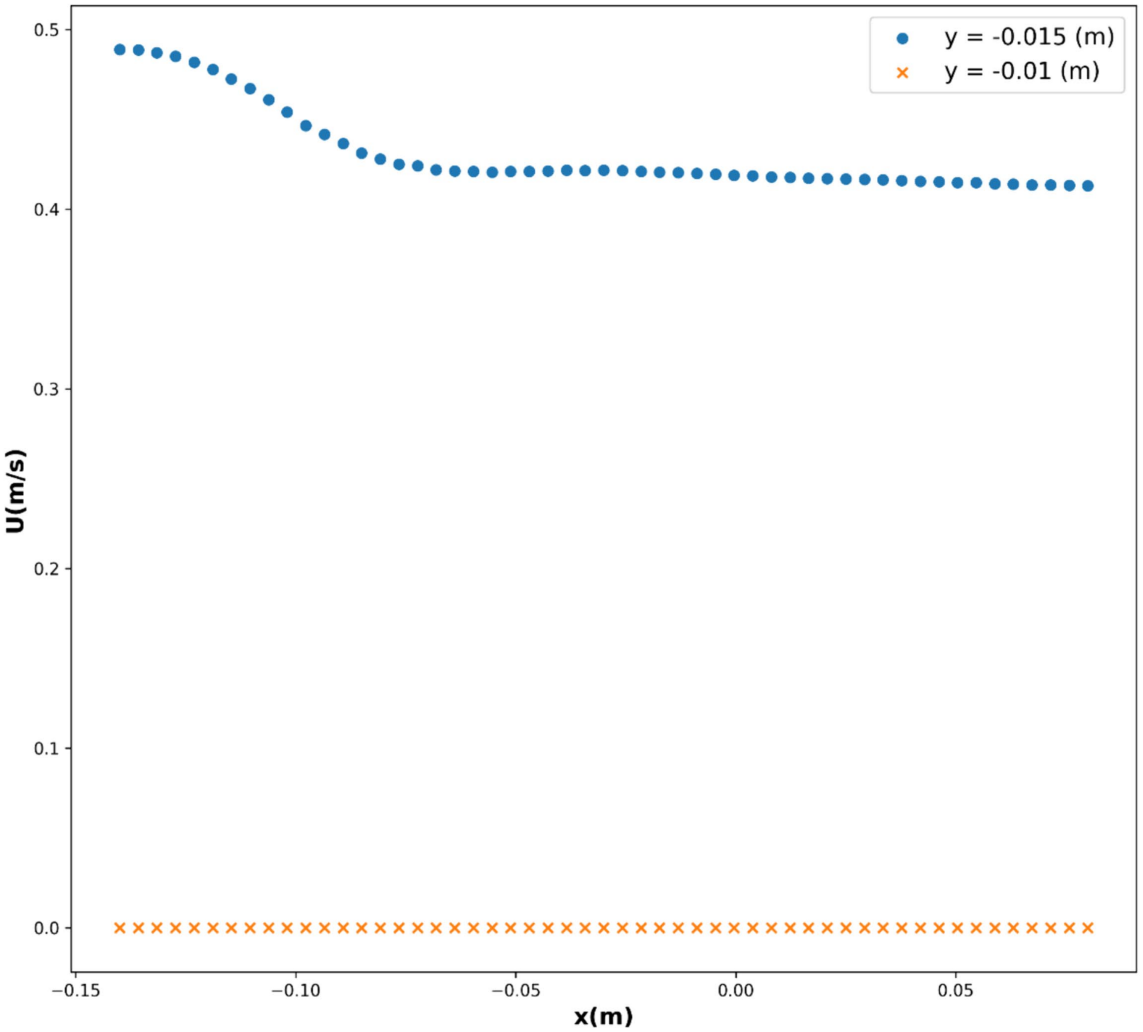
Partial dependency of *x*(m) on *U*(m/s).

**Figure 10:**
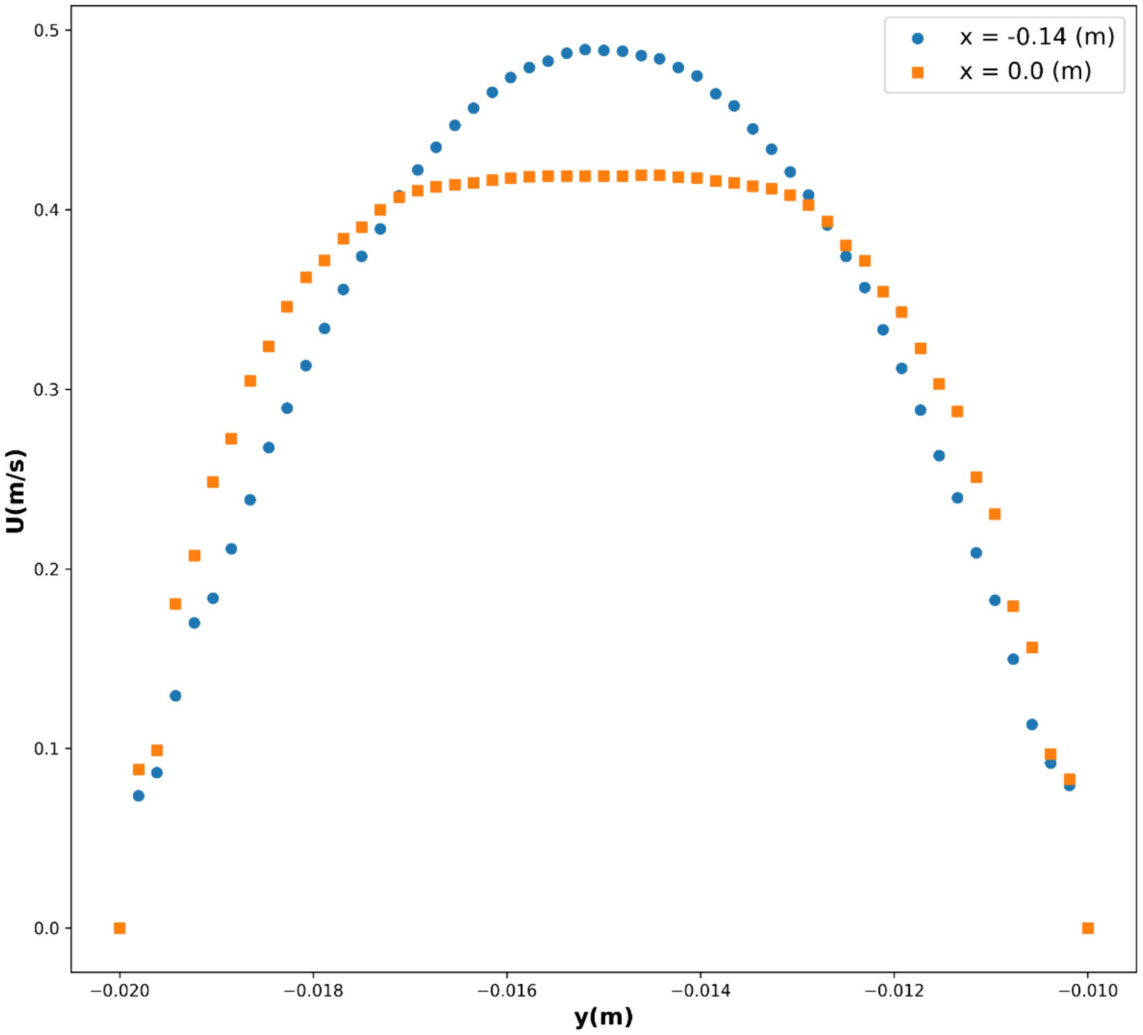
Partial dependency of *y*(m) on *U*(m/s).

**Figure 11:**
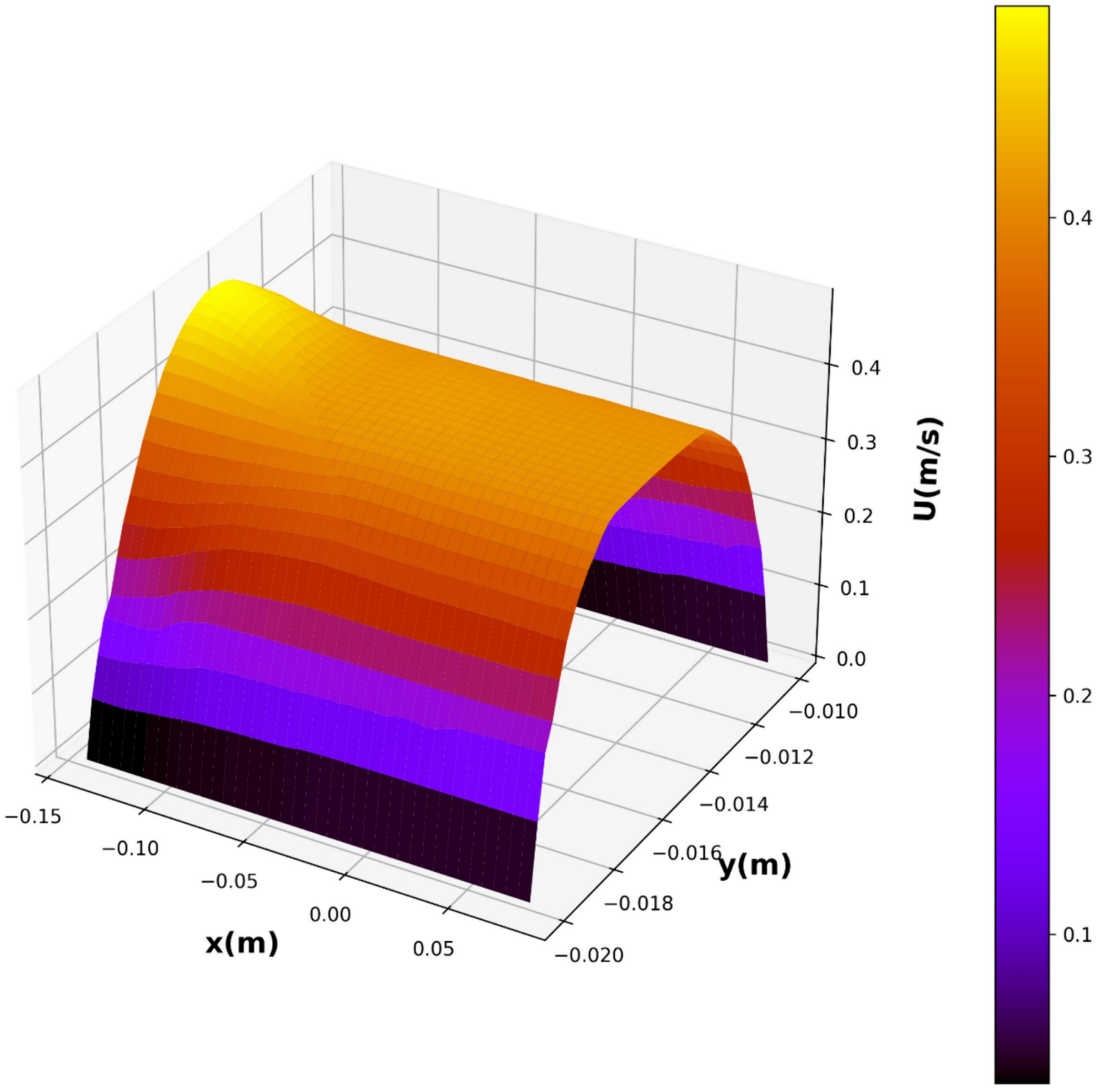
Final 3D prediction surface plot of velocity distribution.

As a result, we chose this model as the best-fit model, with the partial dependencies depicted in Figures 9, 10, and the 3D surface of this model depicted in Figure 11. The parabolic velocity profile can be observed in radial direction which is due to the imposed boundary conditions as well as the effect of viscous forces on the flow pattern. It should be noted that we considered continuum medium for the fluid flow containing the nanoparticles, however future study can develop two-phase flow models for considering the existence of solid particles and their influence on the flow pattern.

## 4 Conclusion

The main aim of the current study was to develop hybrid models for simulation of blood flow through vessel containing magnetic nanoparticles. The role of nanoparticles is the carrier for cancer drug to reach the target. The modeling strategy was conducted in two steps, i.e., CFD simulations followed by ML modeling. Based on the dataset containing variables *x*(m), *y*(m), and *U*(m/s), the aim of this research was to develop an ensemble model using the AdaBoost algorithm for predicting the velocity, represented by *U*(m/s). Three base models, namely DT, KNN, and MLP, were utilized in conjunction with AdaBoost to enhance predictive accuracy.

The results obtained from the AdaBoost ensemble model in combination with each of the base models were highly promising. The ADA-DT model demonstrated a highly notable *R*^2^ value of 0.99783 and a remarkably low RMSE of 5.2893 × 10^−3^. In a similar vein, the ADA-KNN model demonstrated a noteworthy *R*^2^ score of 0.98524, accompanied by an RMSE of 1.3291 × 10^−2^. The ADA-MLP model demonstrated exceptional performance, achieving an *R*^2^ value of 0.99603 and an RMSE of 7.1369 × 10^−3^.

The results of this study underscore the efficacy and dependability of the AdaBoost ensemble model in forecasting velocity using the provided dataset. The high *R*^2^ scores and low RMSE values indicate that the model accurately captures the relationship between the input features [*x*(m) and *y*(m)] and the output variable [*U*(m/s)]. This showcases the potential of the ensemble approach in improving prediction accuracy over individual base models.

It is worth mentioning that the hyper-parameter optimization of the ensemble model was conducted using the BAT optimization algorithm. This approach ensured that the model parameters were fine-tuned to achieve optimal performance. The utilization of such advanced optimization techniques further enhances the credibility and robustness of the findings.

In conclusion, this study successfully developed an AdaBoost ensemble model incorporating base models such as DT, KNN, and MLP to predict velocity based on the provided dataset. The impressive performance of the ensemble model, as evidenced by high *R*^2^ scores and low RMSE values, underscores its efficacy in accurately estimating velocity. The utilization of the BAT optimization algorithm for hyper-parameter optimization adds a layer of sophistication to the research methodology.

## Data availability statement

The original contributions presented in the study are included in the article/supplementary material, further inquiries can be directed to the corresponding author.

## Author contributions

RA: Data curation, Investigation, Software, Writing – original draft. MA: Formal analysis, Methodology, Validation, Writing – original draft. JA: Project administration, Resources, Software, Writing – original draft.
